# Consumption of Goats’ Milk Protects Mice From Carbon Tetrachloride-Induced Acute Hepatic Injury and Improves the Associated Gut Microbiota Imbalance

**DOI:** 10.3389/fimmu.2018.01034

**Published:** 2018-05-15

**Authors:** Jiachao Zhang, Zhaoxia Wang, Dongxue Huo, Yuyu Shao

**Affiliations:** ^1^College of Food Engineering and Nutritional Science, Shaanxi Normal University, Xi’an, China; ^2^College of Food Science and Technology, Hainan University, Haikou, China

**Keywords:** animal milk, gut microbiome, hepatoprotective effect, acute liver injury, carbon tetrachloride

## Abstract

Drugs used to treat liver diseases have serious side effects; it is important to search for safe functional foods with hepatoprotective functions and few side effects. In this study, potential hepatoprotective effects of goats’ milk and cows’ milk on mice with CCl_4_-induced acute hepatic injury were evaluated. We also elucidated the role of goats’ and cows’ milk on the regulation of CCl_4_-induced gut microbiota imbalance. In mice with liver damage induced by CCl_4_, administration of goats’ milk for 7 days prior to injection of CCl_4_ had beneficial effects on the indicators of liver damage within 1 day: the area of liver necrosis was small; activity of alanine transaminase (ALT) and aspartate transaminase (AST) and expression of the genes *CYP2E1* and *TNF-*α were lower than that of model group of mice. By 7 days after CCl_4_ injection, there were no significant differences in liver damage indicators (ALT, AST, malondialdehyde, superoxide dismutase, and glutathione) between the goats’ milk group, which continued to receive goats’ milk, and the untreated control group of mice showing that goats’ milk continued to protect against liver damage. Throughout the entire experiment, the community of gut microbes from mice in the goats’ milk treatment was more similar to the untreated control group than to the cows’ milk group and the model group, indicating that intake of goats’ milk prior and post-CCl_4_ injection effectively prevented and alleviated the intestinal microbial disorder that caused by CCl_4_ in mice. Our research suggests that goats’ milk could be developed as a potential functional food to prevent/protect against liver injury.

## Introduction

Liver disease is a major cause of mortality and morbidity worldwide. Liver function can be impacted by gut function and *vice versa* (1). Following liver injury, the secretion of bile can be altered by the gut–liver axis; this reduces blood supply and peristalsis in the intestine, resulting in disruption of the intestinal mucosa and disturbance of the gut microbiota (2). Gut secretions such as hormones, inflammatory mediators, and digestive absorption products also affect liver function directly (3). The microbiome of the gut, especially in the large intestine, comprised numerous microorganisms that play an important role in food digestion and also in other important processes within the organism (4). Evidence suggests that some gut-derived microbial components activate the inflammatory cascade of immune cells in the liver and regulate the function and response of liver parenchymal cells (5). Imbalance in the gut microbiota can result in severe systemic infections, and alterations in microbial composition and abundance are critically influential factors in hepatic dysfunction (6). Severity of liver disease can be directly related to the severity of dysbiosis of gut microbiota. Sustaining a dynamic balance in gut microbiota could maintain a healthy state, while an imbalance in gut microbiota may promote liver injury. Thus, it is important to fully elucidate the relationship between gut microbiota and acute liver injury that could lead to chronic liver disease.

Some drugs that are used for the treatment of liver diseases have side effects (7). Therefore, it is essential to search for potential functional foods that prevent/protect against liver injury but have no (or few) side effects. Milk is rich in nutrients, especially goats’ milk, which is easier to digest and absorb than cows’ milk due to its smaller casein micelles and fat globules, and the high levels of medium- and short-chain fatty acids (SCFAs) (8). Consumption of goats’ milk can protect cells from injury and has been used as a treatment for hepatic adipose infiltration in children (9). This indicates that goats’ milk may have potential hepatoprotective qualities that should be further investigated.

Carbon tetrachloride (CCl_4_)-induced hepatic injury in animal models has been used to explore the potential for natural compounds to protect the liver against damage (10). CCl_4_ transforms into a trichloromethyl radical that reacts with molecular oxygen to form a highly toxic trichloromethyl peroxyl radical; this free radical disrupts polyunsaturated fatty acids in membrane lipids causing the membrane structures to rupture, leading to the disruption of cell energy processes and the synthesis of protein, which leads to lipid peroxidation in liver cells (11). Furthermore, the community structure of the gut microbiota is altered following oral administration or intraperitoneal injection of CCl_4_ (12, 13). This means that CCl_4_ directly induces hepatic injury, while simultaneously modulating the gut microbiota. Mazagova et al. and Wang et al. were the first to demonstrate the protective role of gut microbiota against CCl_4_-induced chronic liver injury in germ-free mice (14); they demonstrated that commensal microbiota were important for maintenance of liver health. Subsequent studies have also proven the beneficial effect of gut microbiota on hepatic function (15). Changes in the gut microbiota could result in changes in liver function. An important component of the gut microbiota is probiotics (e.g., *Lactobacillus, Bifidobacterium, Clostridium*, and *Saccharomyces* species), which are also known to alleviate the symptoms of CCl_4_-induced hepatic injury (12, 16). Previous study showed that goats’ milk had beneficial effects on rats with hepatotoxicity induced by the drugs isoniazid, rifampicin, and pyrazinamide (17). In this study, the preventative and protective effects of goats’ milk and cows’ milk, administered prior to and post the use of on CCl_4_ to induce acute liver injury mice, were investigated. We also used metagenomics-wide high-throughput sequencing of the V3–V4 regions of the 16S rRNA gene to study the beneficial roles of the milk on the regulation of CCl_4_-induced gut microbiota imbalance. Our research suggests that goats’ milk could be developed as a potential functional food to prevent/protect against liver injury and provides the preliminary data necessary for future in-depth studies on the relationships amongst goat milk, gut microbiota, and acute liver injury.

## Materials and Methods

### Mice and Experimental Protocol

C57BL/6 male mice (12 weeks old) were allowed to acclimate to the animal facility for 1 week before starting the experiment. Mice were maintained in individual ventilated cages in climate-controlled rooms (12 h light/dark cycles; 20–24°C; 45–55%RH; individual filter-sterilized air) with access to food and water *ad libitum*. All food, water, and experimental equipment used were sterilized or sanitized. The animals received humane care from trained staff, and all experiments were performed in accordance with the guidelines of the Shaanxi Normal University Scientific Ethics Committee.

**Figure 1:**
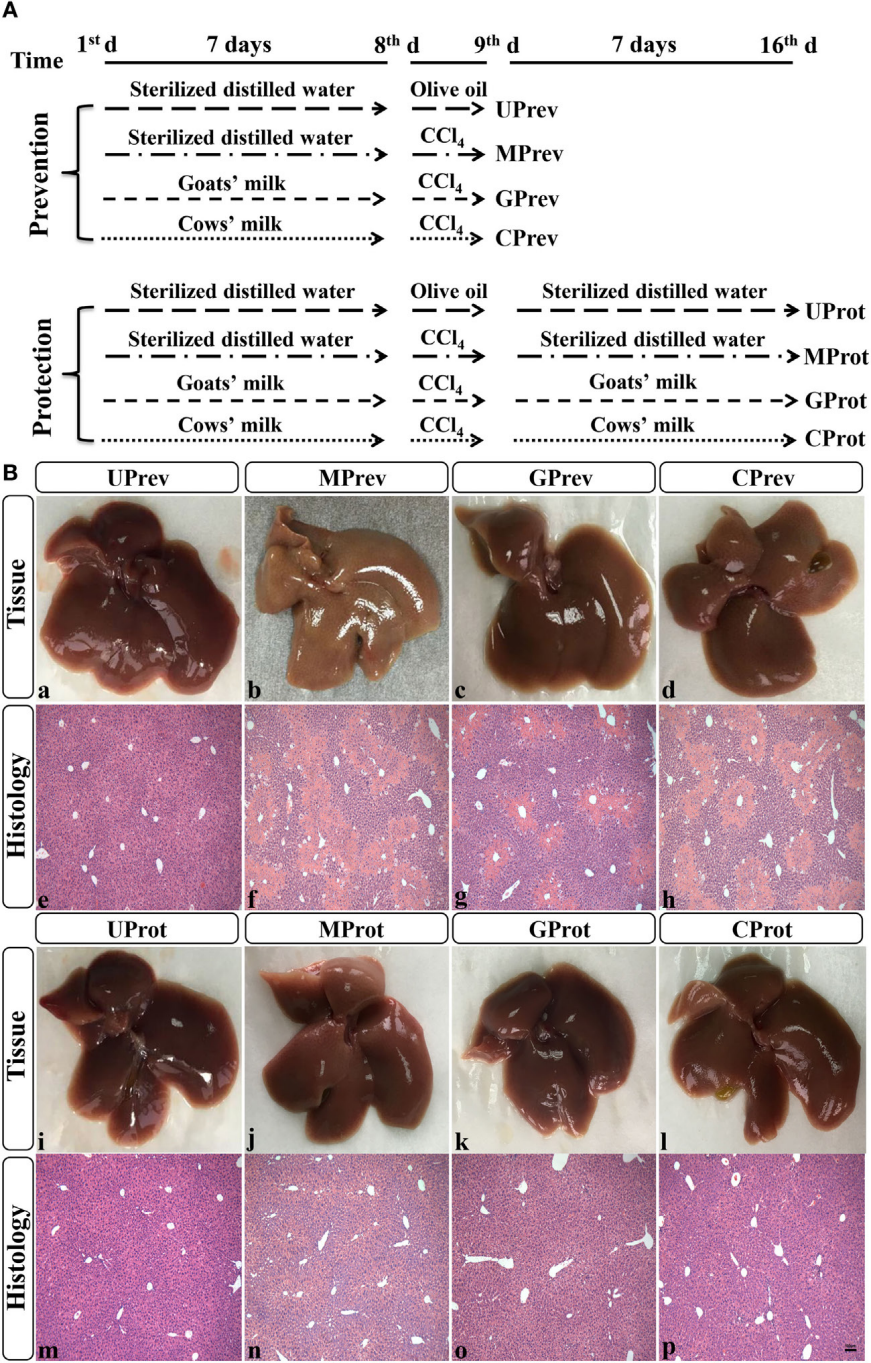
Experimental design **(A)** and histological analysis of livers from the eight different treatment groups of mice (see Figure 1A) **(B)** (1). Liver samples taken 1 day after administration of CCl_4_ (to determine preventive effects of milk consumption on induced liver injury) from untreated control mice UPrev (a), model mice MPrev (b), mice receiving goats’ milk GPrev (c), and mice receiving cows’ milk CPrev (d) and 8 days after administration of CCl_4_ (to determine protective effects of continued milk consumption on induced liver injury) from untreated control mice UProt (i), model mice MProt (j), mice receiving goats’ milk GProt (k), and mice receiving cows’ milk CProt (l) (2). Liver histology [hematoxylin and eosin (H&E)] of UPrev (e), MPrev (f), GPrev (g), CPrev (h), UProt (m), MProt (n), GProt (o), and CProt (p) mice. Scale bar: 100 µm for e–h and m–p.

After 1 week of acclimatization, the mice were divided randomly into eight groups, each comprised six mice (48 in total) and fed the same diet throughout the experimental period. Four groups were used to evaluate the preventative effects of milk consumption for 7 days prior to challenge with CCl_4_, and the other four groups to evaluate the protective effects of continued milk consumption in the 7 days following challenge with CCl_4_. The eight groups were set up at the same time and run alongside each other; the experimental design is fully described in Figure 1A. For each part of the experiment (preventive effects and protective effects), there was an untreated control group of mice (U; receiving only food and water), a model group (M; receiving CCl_4_ on day 7), a goats’ milk group (G; receiving CCl_4_ on day 7 and goats’ milk daily throughout), and a cows’ milk group (C; receiving CCl_4_ on day 7 and cows’ milk daily throughout). On day 8 of the experiment (1 day after injection of CCl_4_ in the M, G, and C groups), the first four groups of mice, designated as UPrev, MPrev, GPrev, and CPrev, were sacrificed and assessed for the preventive role of milk on liver injury prior to challenge with CCl_4_. After a further 7 days, the remaining groups were sacrificed and assessed for the protective role of continued administration of milk against liver injury following challenge with CCl_4_ and these were designated as UProt, MProt, GProt, and CProt, respectively.

#### Evaluating the Preventive Role of Milk on CCl_4_-Induced Liver Damage

From days 1–7, mice in treatment groups UPrev and MPrev received 10 mL/kg body weight sterilized distilled water by intra-gastric administration twice a day; mice in treatment groups GPrev and CPrev received 10 mL/kg body weight of commercial UHT-goats’ milk and 10 mL/kg body weight of commercial UHT-cows’ milk, respectively, again twice a day by intra-gastric administration.

On the 8th day, mice in the UPrev group received an intraperitoneal injection of 1 mL/kg body weight olive oil; mice in groups MPrev, GPrev, and CPrev received an intraperitoneal injection of 1 mL/kg body weight CCl_4_ (1:4, v/v, in olive oil). After 24 h (day 9), all mice were anesthetized with isoflurane gas, and under aseptic conditions, laparotomies performed via a midline incision and samples collected from the colon for gut microbiota evaluation. Blood samples were taken and the livers harvested, frozen in liquid nitrogen, and stored at −80°C for further analysis.

#### Evaluating the Protective Effects of Milk on CCl_4_-Induced Liver Damage

Mice in the other four groups were used to evaluate the protective effects of continued milk consumption and were treated the same as the mice in the preventive experiment, until day 9 but then, rather than being sacrificed, UProt, MProt, GProt, and CProt mice continued to receive the sterilized distilled water, goats’ milk, or cows’ milk for a further 7 days. On the 16th day, all remaining mice were sacrificed and samples were collected as described earlier.

### Histological Analysis

Samples from the left lobe of the liver were fixed in 4% paraformaldehyde (4°C), dehydrated in graded alcohol, and embedded in paraffin wax. Embedded tissues were cut into 4 µm thick sections and stained with hematoxylin and eosin for morphological analysis.

### Alanine Transaminase (ALT) and Aspartate Transaminase (AST) Analyses

ALT and AST are mainly distributed in liver cells, and when the membranes of liver cells are damaged or undergo necrosis, these enzymes enter the serum in large quantities. Determination of serum or plasma enzyme activity can sensitively reflect the extent of liver injury (18). Serum was collected by centrifugation (2,000 × *g* for 15 min, 4°C). ALT and AST levels were determined using the commercial ALT Activity Assay Kit (MAK052) and the AST Activity Assay Kit (MAK055) according to the manufacturer’s instructions (Sigma-Aldrich, Inc.). Each sample was assayed three times.

### Malondialdehyde (MDA), Superoxide Dismutase (SOD), and Glutathione (GSH) Analyses

Samples of liver were weighed and homogenized for 1 min in phosphate buffer (pH 7.4). The homogenates were centrifuged at 2,000 × *g* and 4°C for 20 min, and the supernatant was collected carefully. MDA, SOD, and GSH concentrations in the supernatants were determined using mouse MDA, SOD, and GSH ELISA kits according to the manufacturer’s instructions (MDA, ml931407; SOD, ml643059; GSH, ml643115; Shanghai Enzyme-linked Biotechnology Co., Ltd). Each sample was assayed three times.

### Quantitative RT-PCR Assay

We measured the mRNA expression levels of the genes for the key enzymes CYP2E1, which influences the activation of CCl_4_ *in vivo*, and TNF-α, which is a pro-inflammatory cytokine. Total RNA was extracted from 50 mg samples of liver tissue using TRIzol^®^ according to the manufacturer’s protocol. Sample RNA was transcribed into cDNA using the SuperScript III RT-PCR kit, and then quantitative PCR was performed in a fluorescent temperature cycler with SYBR Green and specific primers for each of the genes. Each reaction was repeated independently at least three times. Glyceraldehyde-3-phosphate dehydrogenase (GAPDH) was amplified as an internal control. The primer sequences used for each gene are listed in Table 1. pcr array data were calculated using the 2^−(ΔΔCt)^ method.

**Table 1:** PCR primers used in this research.

Gene	Forward	Reverse
*CYP2E1*	TTTCCCTAAGTATCCTCCGTGAC	CGTAATCGAAGCGTTTGTTGA
*TNF-*α	TGAGGTCAATCTGCCCAAGT	CTGAGCCATAATCCCCTTTCTA
*GAPDH*	GGTTGTCTCCTGCGACTTCA	TGGTCCAGGGTTTCTTACTCC
*16S rRNA* gene (V3–V4 regions)	ACTCCTACGGGAGGCAGCA	GGACTACHVGGGTWTCTAAT

### Metagenomic DNA Extraction and High-Throughput Sequencing of V3–V4 Regions of 16S rRNA Gene

Extraction and analysis of metagenomic DNA from the microbiome present in colon samples was performed using our previously described methods (19). The V3–V4 regions of the 16S rRNA gene were amplified using specific primers (forward primer 338F and reverse primer 806R; Table 1) (20). PCR products were purified using the Qiagen Gel Extraction Kit. A TruSeq^®^ DNA PCR-Free Sample Preparation Kit was used to construct the DNA library. The DNA library was quantified using a Qubit Fluorometer and an Agilent Bioanalyzer 2100; sequencing was done using an Illumina HiSeq 2500 System.

### Bioinformatics Analysis of Sequence Data

The 250 bp paired-end reads were generated through sequencing; the bioinformatics analysis we used for these sequence data has been fully described previously (8, 19, 20). Briefly, Qiime pipeline (v1.7.0) was used to filter out low-quality tags. Uparse software (v7.0.1001) was used to cluster effective tags to the operational taxonomic unit (OTU) based on 97% sequence similarity of sequences. Representative OTUs with the high frequency of occurrence were selected and annotated for taxonomic information using the Mothur method and SSUrRNA database in SILVA with a threshold of 0.8–1, to obtain community compositions at different taxonomic levels (phylum and genus). Multiple sequence alignments were performed using MUSCLE software to study phylogenetic relationships amongst different OTUs and the predominant bacteria in the gut microbiota.

Alpha diversity was determined as a measure of the diversity of the microbial community within a sample; Shannon index was calculated by Qiime to measure the alpha diversity of the gut microbiota (8, 19, 20). Beta diversity is a comparative analysis of microbial community composition and complexity between sample pairs (8, 19, 20); Weighted UniFrac distances between microbial communities from the colon samples were calculated by Qiime to study beta diversity metrics (8, 19, 20). Based on the UniFrac distances between gut microbial communities, the samples located in the first principal coordinates analysis at different time points were used to fit the curve of changes in microbial community structure and to dynamically analyze differences between the treatment groups in their microbial community structure; this allowed us to identify groups with community structures closest to the untreated control group and thus reveal the treatments providing the best prevention and mitigation effects in the CCl_4_-induced mice. Phylogenetic Investigation of Communities by Reconstruction of Unobserved States (PICRUSt) was used to predict the function of the 16S rRNA gene based on the sequencing data (21). *Z* scores for each of the specific metabolic pathway in pairwise group were calculated using R software. Relationships amongst goats’ milk, different genera, metabolisms, and liver damage indicators were calculated using the Spearman Rank Correlation Coefficient and visualized as a network in Cytoscape software (v3.2.1). R software and GraphPad Prism were used for plotting in this study.

### Statistical Analysis

Analysis of differences between two or more groups was performed using the non-parametric Wilcoxon rank-sum test and the Kruskal–Wallis *H* test. The data and error bars are presented as mean ± standard error of the mean. Statistical significance was assumed when the *P* value was less than 0.05. All statistical analysis was performed in R software.

### Nucleotide Sequence Accession Number

The high-throughput sequencing data reported in this study were deposited in the NCBI database, and the accession number is PRJNA437795.

## Results

### Histological Analysis

#### Preventive Role of Milk on CCl_4_-Induced Liver Damage

Liver tissue appeared reddish brown and shiny in the UPrev mice 24 h after olive oil injection (Figure 1B-a); the hepatic lobule structure was clear and complete, the liver cells were large, the nuclei were round and located in the middle of the cell, and there was no degeneration, necrosis, or infiltration of inflammatory cells (Figure 1B-e). However, in the MPrev mice, the liver tissue was pale and infiltrated with white spots that indicated injury inside the hepatic cells (Figure 1B-b); large areas of necrosis were apparent in the central parts of the hepatic lobules, and the necrotic area was characterized by fragmentation, dissolution, and disappearance of the nucleus. There was a necrotic band between the two central veins, and normal liver tissue between the necrotic areas (Figure 1B-f). Liver tissues from the GPrev mice (Figure 1B-c) were slightly paler than tissue from the UPrev mice but with fewer scattered white spots compared with the MPrev mice. There was a little necrosis of the hepatic lobule in liver tissue from GPrev mice (Figure 1B-g); the necrotic area was intermediate in size between the UPrev mice and the CPrev mice (Figure 1B-d,h). This indicated that GPrev mice had the least liver injury following CCl_4_ challenge, showing that goats’ milk had a protective effect against acute liver injury.

#### Protective Role of Milk on CCl_4_-Induced Liver Damage

Eight days after CCl_4_ injection, the liver tissue and hepatic lobule structure of MProt mice (Figure 1B-j,n), GProt mice (Figure 1B-k,o), and CProt mice (Figure 1B-l,p) were similar to those from the UProt mice (Figure 1B-i,m). The necrotic area of liver tissue disappeared, and the structure of the liver lobule returned to normal, indicating that the tissue had been repaired.

### ALT and AST Analyses

#### Preventive Role of Milk on CCl_4_-Induced Liver Damage

**Figure 2:**
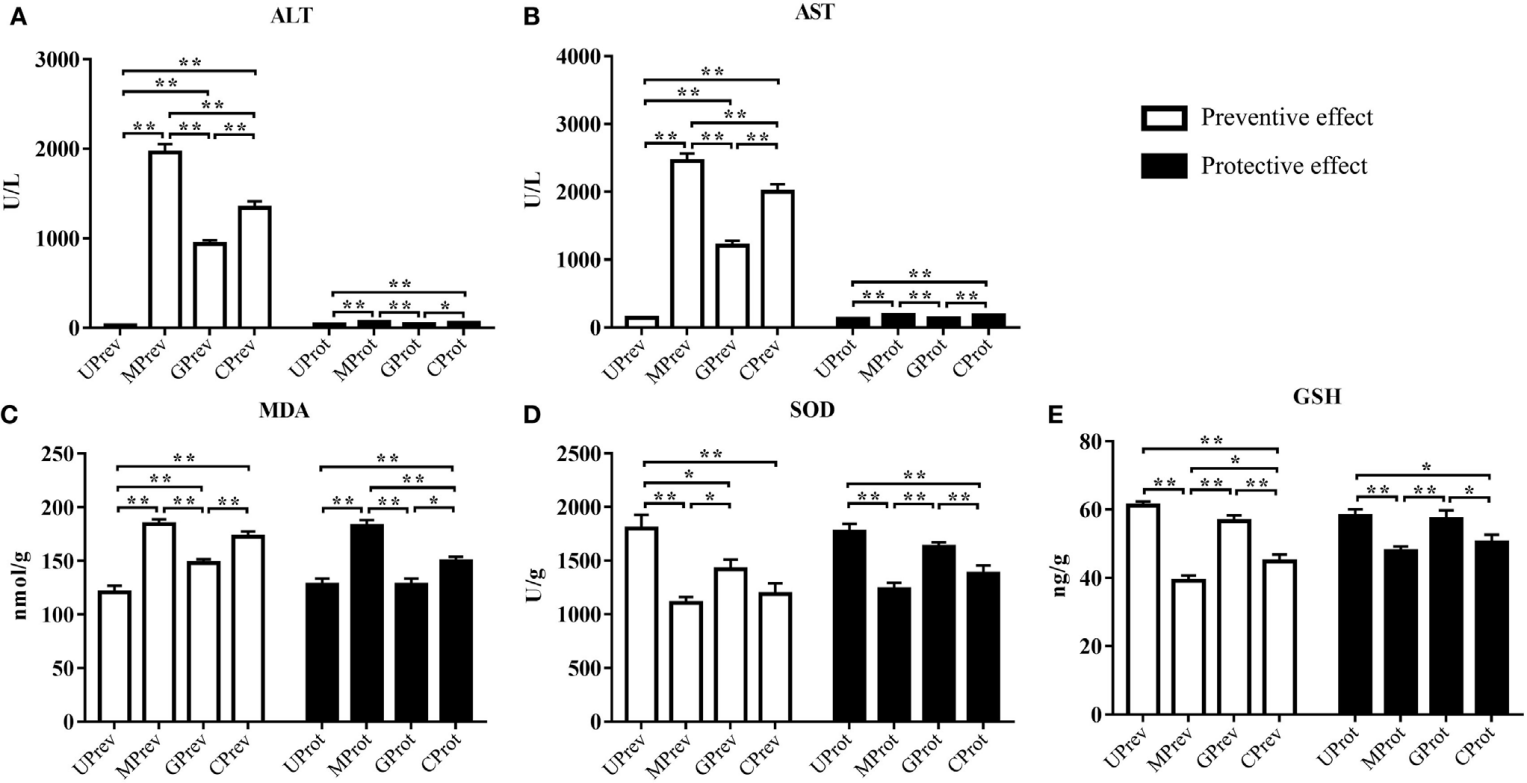
Levels of five biochemical indices in samples from mice in the eight different treatment groups (see Figure 1A). Samples were taken 1 day after administration of CCl_4_ (to determine preventive effects of milk consumption on induced liver injury) from: untreated control mice (UPrev), model mice (MPrev), mice receiving goats’ milk (GPrev), and mice receiving cows’ milk (CPrev) and 8 days after administration of CCl_4_ (to determine protective effects of continued milk consumption on induced liver injury) from untreated control mice (UProt), model mice (MProt), mice receiving goats’ milk (GProt), and mice receiving cows’ milk (CProt). The indices were as follows: alanine transaminase (ALT) **(A)**, aspartate transaminase (AST) **(B)**, malondialdehyde (MDA) **(C)**, superoxide dismutase (SOD) **(D)**, and glutathione (GSH) **(E)**. The data and error bars are presented as mean ± SEM. *P < 0.05; **P < 0.01.

Twenty-four hours after administration of CCl_4_, ALT levels in serum from MPrev and GPrev mice were 72 and 35 times higher than that of UPrev mice (Figure 2A). AST levels in serum from MPrev and GPrev mice were 17 and 9 times higher than that of UPrev mice (Figure 2B). All differences between the groups presented above were statistically significant (*P* < 0.01).

#### Protective Role of Milk on CCl_4_-Induced Liver Damage

Eight days after injection of CCl_4_, ALT and AST levels in serum from MProt mice were 1.8 and 1.4 times higher than in serum from UProt mice (*P* < 0.01), respectively. However, there were no significant differences in ALT and AST levels between GProt and UProt mice (*P* > 0.05). This shows that peak liver injury occurred 24 h after injection of CCl_4_ and that the degree of liver damage in the goats’ milk group of mice was significantly lower than in the model group and the cows’ milk group, which paralleled the histopathological findings. This indicates that goats’ milk protects against acute CCl_4_-induced liver damage and could accelerate the recovery of damaged tissue.

### MDA, SOD, and GSH Analyses

#### Preventive Role of Milk on CCl_4_-Induced Liver Damage

Twenty-four hours after injection of CCl_4_, MDA levels in the liver tissues of MPrev mice were significantly higher than in the liver tissues of UPrev and GPrev mice (*P* < 0.01; Figure 2C); MDA levels were significantly higher in the liver tissues of GPrev mice compared with UPrev mice (*P* < 0.01; Figure 2C). SOD and GSH levels in liver tissues of MPrev mice were significantly lower than those in UPrev mice (*P* < 0.01; Figures 2D,E), but there was no significant difference in GSH levels in GPrev mice compared with UPrev mice (*P* > 0.05).

#### Protective Role of Milk on CCl_4_-Induced Liver Damage

MDA levels in the liver tissues of MProt mice were significantly higher than in the liver tissues of UProt and GProt mice (*P* < 0.01; Figure 2C). There was no significant difference between MDA levels in GProt and UProt mice (*P* > 0.05). SOD and GSH levels were significantly lower in MProt mice than in UProt mice (*P* < 0.01; Figures 2D,E), but there were no significant differences in SOD and GSH levels between GProt mice and UProt mice (*P* > 0.05).

### Quantitative RT-PCR Assay of *CYP2E1* and *TNF*-*α*

#### Preventive Role of Milk on CCl_4_-Induced Liver Damage

**Figure 3:**
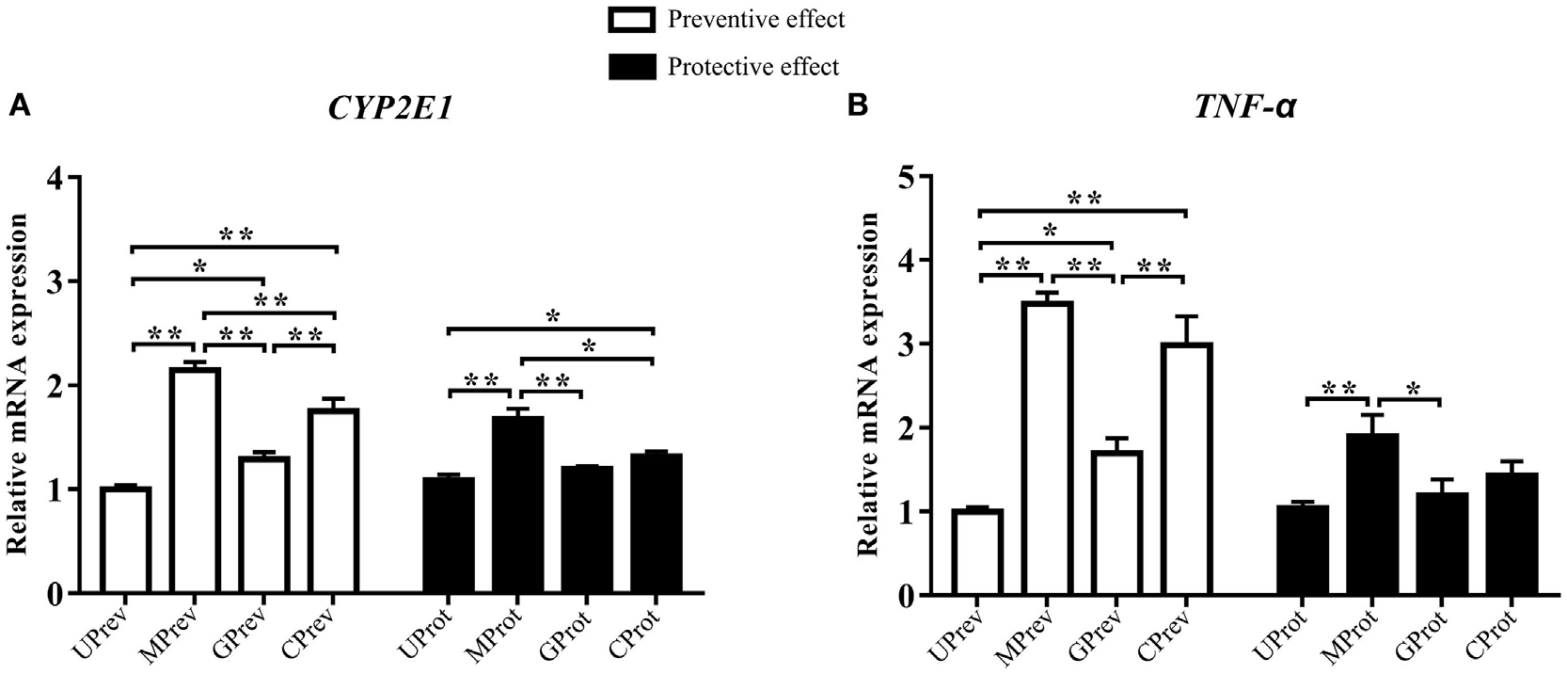
Relative expression levels of two key genes *CYP2E1* **(A)** and *TNF-*α **(B)** in liver tissue from mice in the different treatment groups (see Figure 1A). Samples were taken 1 day after administration of CCl_4_ (to determine preventive effects of milk consumption on induced liver injury) from: untreated control mice (UPrev), model mice (MPrev), mice receiving goats’ milk (GPrev), and mice receiving cows’ milk (CPrev) and 8 days after administration of CCl_4_ (to determine protective effects of continued milk consumption on induced liver injury) from untreated control mice (UProt), model mice (MProt), mice receiving goats’ milk (GProt), and mice receiving cows’ milk (CProt). The data and error bars are presented as mean ± SEM. *P < 0.05; **P < 0.01.

Twenty-four hours after the administration of CCl_4_, the relative expression levels of *CYP2E1* were 2.2, 1.3, and 1.8 times higher in the liver tissues of MPrev, GPrev, and CPrev mice, respectively, than those in the liver tissues of UPrev mice (*P* < 0.05; Figure 3A). The relative mRNA expression levels of *TNF-*α in liver tissues of MPrev, GPrev, and CPrev mice were 3.5, 1.7, and 3.0 times higher than those in UPrev mice, respectively (*P* < 0.05, Figure 3B).

#### Protective Role of Milk on CCl_4_-Induced Liver Damage

Eight days after CCl_4_ administration, the relative mRNA expression levels of *CYP2E1* in liver tissues of MProt mice were 1.5 times higher than those in UProt mice (*P* < 0.01), while there were no significant differences in expression between GProt and UProt mice (*P* > 0.05). The results suggest that mRNA expression levels of *CYP2E1* tend to normalize following goats’ milk intervention and that expression was repressed by goats’ milk. The relative mRNA expression levels of *TNF-*α in the liver tissue of MProt mice were 1.8 times higher than those in UProt mice (*P* < 0.01), while expression levels in GProt mice were only 1.1 times higher than those in UProt mice (*P* > 0.05). The results showed that the expression of *TNF-*α tended to normalize in mice following goats’ milk intervention.

### Diversity of Gut Microbiota from Different Mice Treatment Groups

**Figure 4:**
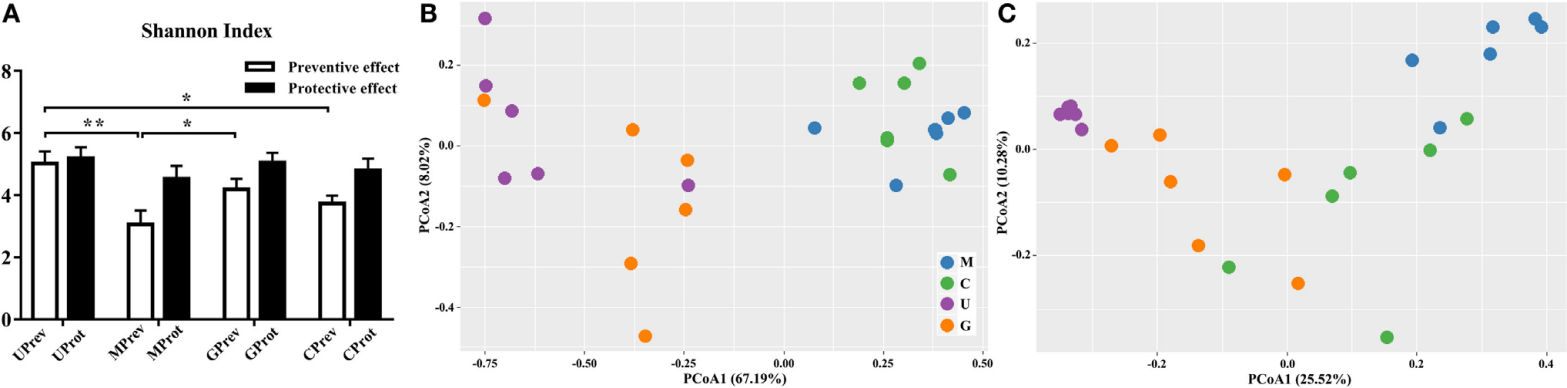
Diversity of the gut microbiota. **(A)** Alpha diversity of the gut microbiota of U, M, G, and C mice as measured by the Shannon index and **(B,C)** beta diversity analyzed using principal components analysis (PCoA) score plots based on weighted UniFrac metrics for the gut microbiota from the eight treatment groups of mice: **(B)** samples taken 1 day after the administration of CCl_4_ (to determine preventive effects of milk consumption on induced liver injury) from untreated control mice (UPrev), model mice (MPrev), mice receiving goats’ milk (GPrev), and mice receiving cows’ milk (CPrev) and **(C)** samples taken 8 days after administration of CCl_4_ (to determine protective effects of continued milk consumption on induced liver injury) from untreated control mice (UProt), model mice (MProt), mice receiving goats’ milk (GProt), and mice receiving cows’ milk (CProt). The data and error bars are presented as mean ± SEM. *P < 0.05; **P < 0.01.

Shannon index was used to determine the alpha diversity of the microbiota in samples. The average value of this index for the microbiota was significantly higher in the UPrev and GPrev groups than in the MPrev group (*P* < 0.05; Figure 4A), indicating that the alpha diversity of the microbiota in UPrev and GPrev was higher than that in MPrev. However, the difference amongst the alpha diversity of the gut microbiota of the four groups at day 16 was not significantly different (*P* > 0.05). The alpha diversities of the microbiota in the MProt, GProt, and CProt groups of mice were all higher than in the values for the MPrev, GPrev, and CPrev groups.

There were significant differences in the beta diversity of gut microbiota between the UPrev and MPrev groups (*P* < 0.05), the GPrev and MPrev groups (*P* < 0.05), the UPrev and CPrev groups (*P* < 0.05), and the GPrev and CPrev groups (*P* < 0.05); no significant differences in beta diversity were detected between the UPrev and GPrev groups (*P* > 0.05), or between the CPrev and MPrev groups (*P* > 0.05). The results were the same for the UProt, MProt, GProt, and CProt data. Principal components analysis showed that the Weighted UniFrac distances between the bacterial communities in the UPrev and GPrev groups were small (Figure 4B). The CProt group tended to be closer to the UProt and GProt groups; the MPrev and MProt groups remained distant from the other groups throughout the experimental period (Figure 4C). Bioinformatics analysis showed that microbial communities of mice in the goats’ milk groups and the untreated control groups were more similar to each other than to communities from mice in the cows’ milk and model groups; this was both in their proximity and their trends in variation. These results indicate that intake of goats’ milk effectively prevented and alleviated intestinal microbial disorder caused by CCl_4_ in mice.

### Taxonomic Annotation of Gut Microbiota From Different Mice Treatment Groups

#### Preventive Role of Milk on CCl_4_-Induced Liver Damage

**Figure 5:**
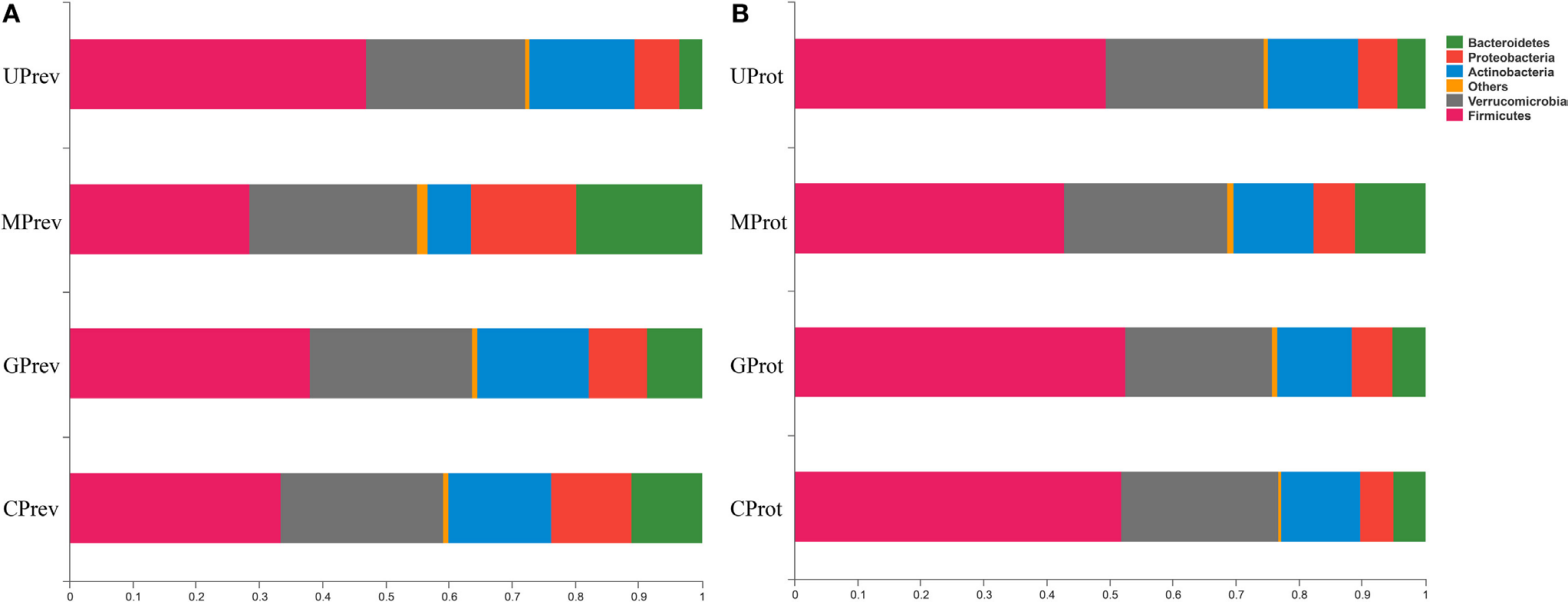
Relative abundance of different phyla in the gut microbiota of mice from the eight treatment groups: **(A)** samples taken 1 day after administration of CCl_4_ (to determine preventive effects of milk consumption on induced liver injury) from: untreated control mice (UPrev), model mice (MPrev), mice receiving goats’ milk (GPrev), and mice receiving cows’ milk (CPrev) and **(B)** samples taken 8 days after administration of CCl_4_ (to determine protective effects of continued milk consumption on induced liver injury) from untreated control mice (UProt), model mice (MProt), mice receiving goats’ milk (GProt), and mice receiving cows’ milk (CProt).

**Figure 6:**
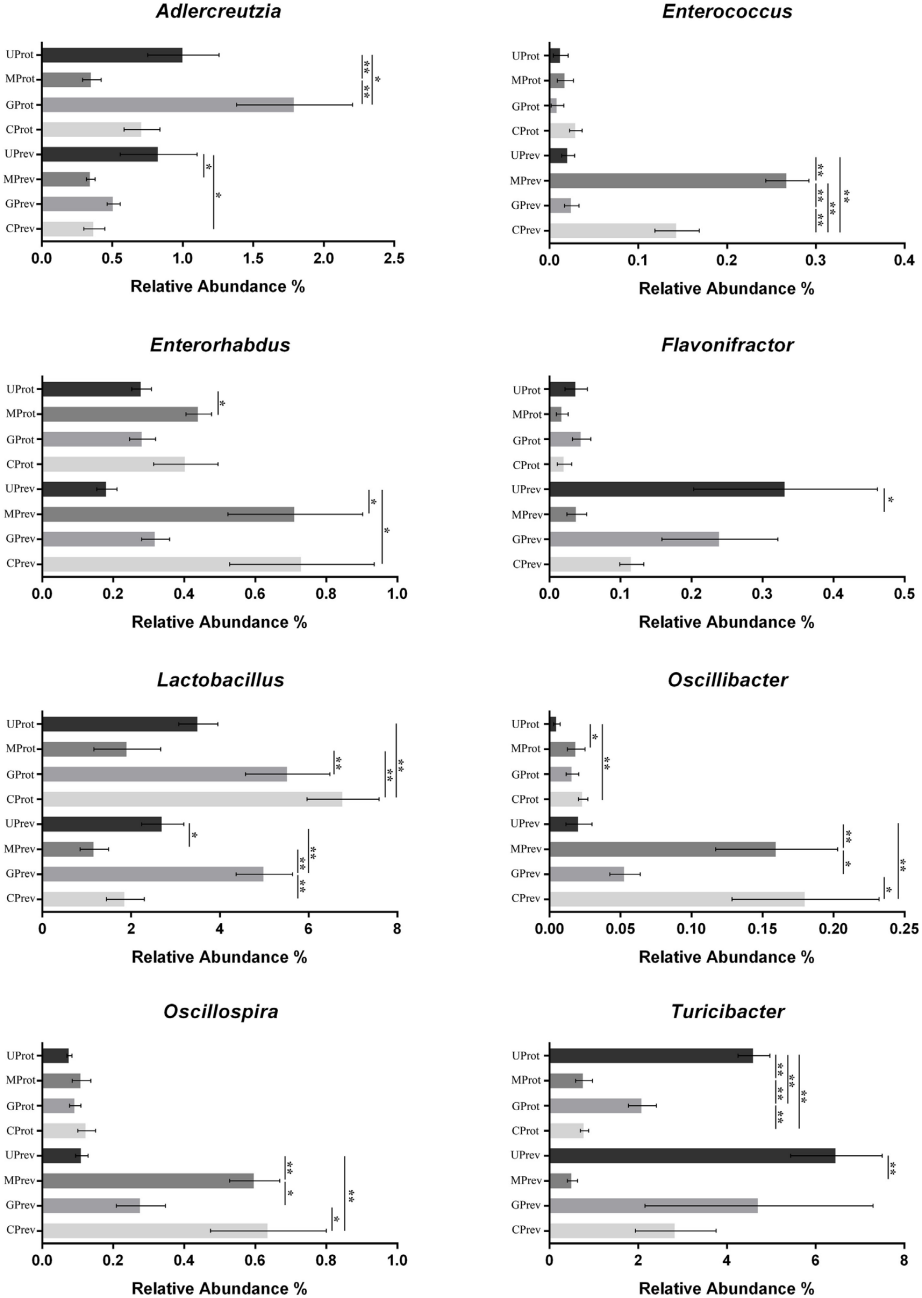
Abundance of the eight microbial genera significantly affected by the experimental treatments, in the gut microbiota of mice from the eight treatment groups: Samples were taken 1 day after the administration of CCl_4_ (to determine preventive effects of milk consumption on induced liver injury) from untreated control mice (UPrev), model mice (MPrev), mice receiving goats’ milk (GPrev), and mice receiving cows’ milk (CPrev) and 8 days after the administration of CCl_4_ (to determine protective effects of continued milk consumption on induced liver injury) from untreated control mice (UProt), model mice (MProt), mice receiving goats’ milk (GProt), and mice receiving cows’ milk (CProt). The data and error bars are presented as mean ± SEM. *P < 0.05; **P < 0.01.

At the phylum level, *Firmicutes, Verrucomicrobia, Actinobacteria, Bacteroidetes*, and *Proteobacteria* were detected in all gut microbiota samples (Figures 5A,B); *Firmicutes* was the predominant phylum, representing more than 43.29% of all bacteria present. Relative abundance of *Firmicutes* was significantly lower in the MPrev group than in the UPrev and GPrev groups (*P* < 0.05), but *Bacteroidetes* were significantly more abundant in the MPrev group than in the UPrev and GPrev groups (*P* < 0.05). At the genus level, significant differences in abundance of different microbial genera were found between the MPrev group and the UPrev group. The genera *Enterococcus, Enterorhabdus, Oscillibacter*, and *Oscillospira* were significantly more abundant in MPrev mice than in UPrev mice (*P* < 0.05), while the genera *Adlercreutzia, Flavonifractor, Lactobacillus*, and *Turicibacter* were more abundant in UPrev mice than in MPrev mice (*P* < 0.05; Figure 6).

#### Protective Role of Milk on CCl_4_-Induced Liver Damage

At the phylum level, there was no significant difference in the relative abundance of *Verrucomicrobia, Actinobacteria*, and *Proteobacteria* amongst the four groups (*P* > 0.05). However, relative abundance of *Firmicutes* was significantly lower in the MProt group than in the UProt and GProt groups (*P* < 0.05), but *Bacteroidetes* were significantly more abundant in the MProt group than in the UProt and GProt groups (*P* < 0.05). At the genus level, differences in distribution and abundance of the six previously mentioned microbial genera (i.e., *Enterococcus, Enterorhabdus, Flavonifractor, Lactobacillus, Oscillibacter*, and *Oscillospira*) were not significantly different in the gut microbiota of the GProt mice and the UProt mice (*P* > 0.05; Figure 6); distribution and abundance of the eight genera in the gut microbiota of CProt mice were more different to the UProt mice than to the GProt mice.

Overall, intraperitoneal administration of CCl_4_ induced an increase in the abundance of *Enterococcus, Enterorhabdus, Oscillibacter*, and *Oscillospira* and a decrease in the abundance of *Adlercreutzia, Flavonifractor, Lactobacillus*, and *Turicibacter*. Disparity amongst the eight genera gradually decreased over time between the MPrev and UPrev groups and between the MProt and UProt groups, indicating that mice are able to self-heal and recover intestinal microbial capability. Intake of goats’ milk could prevent imbalance in the gut microbiota and promote self-healing and recovery.

### Enrichment of Functional Genes and Metabolic Pathways in Intestinal Microflora

The phosphotransferase system (PTS), glyoxylate and dicarboxylate metabolism, fructose and mannose metabolism, peptidoglycan biosynthesis, d-alanine metabolism, and selenocompound metabolism were enriched in the gut microbiota of UPrev mice (Table 2). Furthermore, glyoxylate and dicarboxylate metabolism, flagellar assembly, PTS, peptidoglycan biosynthesis, synthesis and degradation of ketone bodies, selenocompound metabolism, and d-alanine metabolism were enriched in the gut microbiota of GPrev mice. This shows that functional features of the gut microbiota of the GPrev mice and the UPrev mice were similar and different to the gut microbiota of the MPrev. More metabolic pathways were enriched in the goats’ milk group than in the untreated group, including histidine metabolism, cysteine and methionine metabolism, starch and sucrose metabolism, alanine, aspartate, glutamate metabolism, and energy metabolism. Enrichment of these carbohydrate and amino acids metabolisms in the gut microbiota of the goats’ milk group may be as a result of intra-gastric administration and the gut bacteria played important roles in nutrients and energy metabolism.

**Table 2:** Different metabolic pathways found in the gut microbiota of mice from the untreated (UPrev) and model (MPrev) treatment groups.

KEGG pathway	*Z* score	UPrev	MPrev	Detected KO rate	Pathway
ko02060	3.864	1.347	0.828	72.152	Phosphotransferase system
ko00630	3.561	0.538	0.512	71.579	Glyoxylate and dicarboxylate metabolism
ko00051	3.332	1.295	1.062	67.708	Fructose and mannose metabolism
ko00550	2.782	0.914	0.845	72.500	Peptidoglycan biosynthesis
ko00473	2.443	0.145	0.128	100.000	d-Alanine metabolism
ko00450	2.376	0.418	0.391	75.000	Selenocompound metabolism
ko00072	2.236	0.065	0.047	100.000	Synthesis and degradation of ketone bodies
ko00730	2.138	0.549	0.531	75.000	Thiamine metabolism
ko03010	2.055	2.702	2.540	58.042	Ribosome
ko00471	2.031	0.164	0.159	83.333	d-Glutamine and d-glutamate metabolism
ko00983	1.650	0.275	0.270	68.182	Drug metabolism other enzymes
ko03070	−5.681	0.579	0.661	82.432	Bacterial secretion system
ko00620	−5.651	0.916	0.999	83.333	Pyruvate metabolism
ko02020	−4.818	1.145	1.432	64.019	Two-component system
ko00540	−4.296	0.127	0.224	84.211	Lipopolysaccharide biosynthesis
ko02040	−4.180	0.170	0.216	100.000	Flagellar assembly
ko00650	−3.499	0.604	0.636	74.390	Butanoate metabolism
ko00290	−3.389	0.525	0.648	88.235	Valine, leucine, and isoleucine biosynthesis
ko00640	−3.290	0.517	0.528	70.455	Propanoate metabolism
ko02030	−3.122	0.239	0.326	92.308	Bacterial chemotaxis
ko04122	−2.965	0.244	0.274	80.952	Sulfur relay system
ko00633	−2.766	0.067	0.083	88.235	Nitrotoluene degradation
ko00770	−2.696	0.477	0.553	72.222	Pantothenate and CoA biosynthesis
ko00330	−2.677	1.094	1.162	64.179	Arginine and proline metabolism
ko00860	−2.603	0.462	0.623	67.925	Porphyrin and chlorophyll metabolism
ko00780	−2.585	0.096	0.135	78.947	Biotin metabolism
ko00400	−2.100	0.776	0.786	62.500	Phenylalanine, tyrosine, and tryptophan biosynthesis
ko00281	−2.090	0.021	0.040	81.250	Geraniol degradation
ko00260	−2.086	0.794	0.829	63.441	Glycine, serine, and threonine metabolism
ko00910	−1.814	0.674	0.716	66.071	Nitrogen metabolism
ko00040	−1.733	0.486	0.498	63.934	Pentose and glucuronate interconversions

*Amongst them, Z values greater than 1.6 represent metabolic pathways that were enriched in the untreated group, while Z values less than −1.6 represent metabolic pathways that were enriched in the model group*.

**Figure 7:**
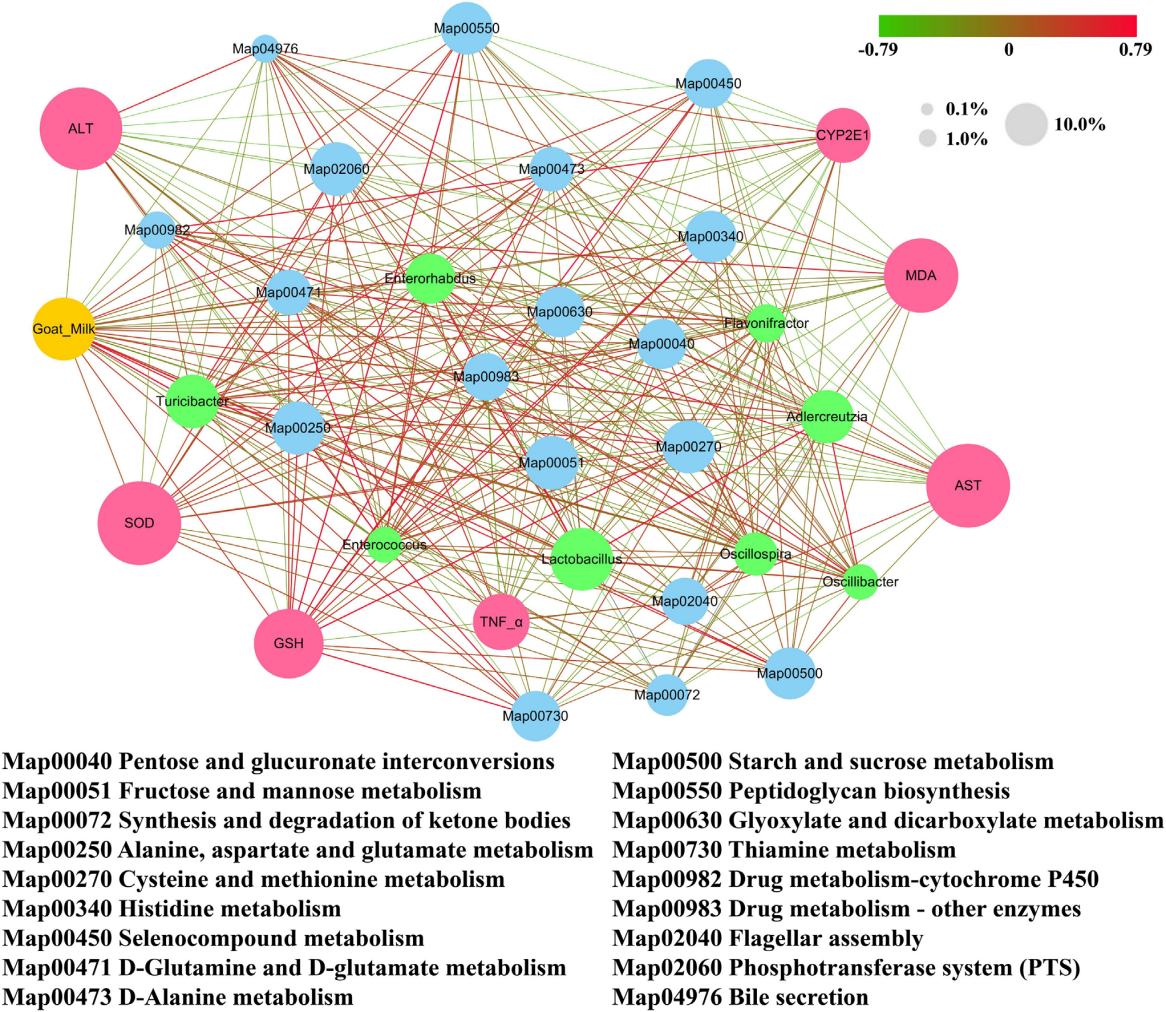
Correlation network established based on goats’ milk, bacterial genera that varied significantly in abundance, G group-enriched metabolisms, and the seven indicators of liver damage. Values of 0.1, 1.0, and 10.0% represent the relative abundance of the same category of elements (i.e., bacterial genera, metabolisms, and liver damage indicators) presented in the network. Positive correlations are connected by red lines (*R* > 0), and negative correlations are connected by gray lines (*R* < 0). The gradually changing color presented in the figure illustrates the degree of the correlation.

We found that metabolism of lipopolysaccharide (LPS) biosynthesis was enriched in model group (*Z* score = −4.296; Table 2). Moreover, a comparison amongst the different groups of mice found that the gut microbiota of the model group was more abundant in drug metabolism-cytochrome P450 and bile secretion than the untreated group (Table S1 in Supplementary Material). Greater enrichment of drug metabolism pathways in the gut microbiota of the model group compared with the other groups may be due to selection of genera that have a functional role in decomposition of the harmful chemical CCl_4_. Low levels of drug metabolism pathways were found in both the goats’ milk group and the untreated group. This may suggest that intake of goats’ milk had some protective effect in mice suffering the challenge of CCl_4_. Correlations amongst goats’ milk, varied significantly amongst bacterial genera, G group-enriched metabolisms, and the seven indicators of liver damage (Figure 7) showed that the G group of mice was positively correlated with *Adlercreutzia* (*R* = 0.35), *Flavonifractor* (*R* = 0.17), *Lactobacillus* (*R* = 0.58), and *Turicibacter* (*R* = *0.71*) but negatively correlated with *Enterococcus* (*R* = −0.22), *Enterorhabdus* (*R* = −0.17), *Oscillibacter* (*R* = −0.28), and *Oscillospira* (*R* = −0.23). The G group of mice was significantly negatively correlated with drug metabolism-cytochrome P450 (*R* = −0.60; *P* < 0.05) and bile secretion (*R* = −0.35; *P* < 0.05).

## Discussion

Liver function can be affected by the food eaten; it is important to search for safe functional foods with potential hepatoprotective abilities. Food can alter the composition and community structure of the gut microbiota, and some foods can promote probiotics; previous studies have demonstrated a beneficial role of commensal gut microbiota and probiotics on liver function. Consequently, we need to take changes in gut microbiota into account when undertaking functional studies of particular foods. In this study, we investigated the hepatoprotective effects of goats’ milk and cows’ milk on mice with CCl_4_-induced acute hepatic injury. We also investigated the regulatory effects of these milks on CCl_4_-induced gut microbiota imbalance. Potential correlation networks amongst milk administration, indicators of acute liver injury, and gut microbiota and their functional features were established.

Histological analysis showed that the area of hepatic cell lobule lesions in the goats’ milk group was smaller than in the model and cows’ milk groups and this was because the expression of *CYP2E1* was inhibited in the liver cells of mice in the goats’ milk group. In fact, CCl_4_ is converted into toxic substances in mice, mainly through activation and induction or over-expression of *CYP2E1*; the CYP2E1 protein is located mainly in the central region of the hepatic lobule, which explains why hepatocyte injury is typically in the centrilobular region after CCl_4_ administration (6). Histological analysis indicated that goats’ milk had beneficial effects on CCl_4_-induced acute hepatic injury; these hepatoprotective abilities of goats’ milk were confirmed by measuring the activity of ALT and AST in serum, which was significantly lower in mice receiving goats’ milk than in the model group of mice.

CCl_4_ is able to induce lipid peroxidation of the liver cell membrane, which is a key factor causing hepatocyte injury (6). Our results showed that ingestion of goats’ milk also prevented the increase in MDA induced by CCl_4_. This would greatly reduce lipid peroxidation injury and prevent a strong inflammatory response (22). Various antioxidant enzymes with high endogenous expression in the liver, such as SOD and GSH, are the major defense mechanisms against reactive oxygen species (23). Current results show that consumption of goats’ milk significantly elevated the activity of SOD and GSH in CCl_4_-damaged livers, indicating the ability of goats’ milk to restore and preserve the activity of these two enzymes.

Kupffer cells are activated by CCl_4_ and could mediate hepatic inflammation by producing TNF-α, which is one of the most potent pro-inflammatory cytokines released by innate immune cells (24). Goats’ milk significantly inhibited the expression of *TNF-*α, suggesting that it plays an important role in reducing the CCl_4_-induced inflammatory cascade in the liver. The mechanism for this protective effect may also be due to the scavenging effect of free radicals, the inhibition of lipid peroxidation, and the enhancement of antioxidant activity. In the goats’ milk group of mice, we observed that the liver tissue was less damaged 24 h after injection of CCl_4_ compared with the other groups receiving CCl_4_; the necrotic area of liver tissue had almost disappeared, and the structure of the liver lobule was restored to normal after 8 days of injection. There are two possible reasons for this phenomenon: the degree of tissue damage was slight and the ability of the tissues to repair was strong. Overall, our findings indicate that goats’ milk has a protective effect against CCl_4_-induced acute hepatic injury in mice, which is achieved by the scavenging of free radicals, inhibiting lipid peroxidation, enhancing the antioxidative defense system, and suppressing cell apoptosis and the inflammatory response of the liver.

Imbalance in the gut microbiota can affect liver function, which could lead to various liver diseases (25). Gut microbiota influence the progression of chronic liver diseases such as alcoholic fatty liver disease (AFLD), cirrhosis, and hepatocellular carcinoma (HCC) (26). AFLD patients have significantly fewer fecal *Bifidobacterium, Lactobacillus*, and *Enterococcus* but more *Escherichia coli* (27). Liver cirrhosis patients have fewer *Faecalibacterium* and *Coprococcus* but more *Fusobacteria* (28). Furthermore, HCC patients have higher proportions of *Helicobacter* (29). Chronic liver diseases develop from acute liver injury, so it is important to understand the relationship between gut microbiota and the severity of acute liver injury. Future studies should always include an accurate assessment of the gut microbiota using sophisticated molecular and microbiological methods. This would facilitate more effective selection of potentially useful functional foods or bacterial isolates that may contribute to the prevention of liver injury. The gut microbiota could be altered through dietary changes or administration of drugs. In this study, mice with CCl_4_-induced acute liver injury had fewer *Adlercreutzia, Flavonifractor, Lactobacillus*, and *Turicibacter* and more *Enterococcus, Enterorhabdus, Oscillibacter*, and *Oscillospira*. Intestinal *Enterococcus* species are known to promote liver disease (30), even though they are lactic acid bacteria. Diets rich in saturated fat can cause the development of a lipogenic liver and is associated with a decrease in the abundance of *Lactobacillus* and an increase in the abundance of *Oscillibacter* in the gut microbiota (31). Gram-negative LPS-producing bacteria, such as *Oscillibacter*, may activate bacterial translocation, leading to liver disease; LPS could also elevate levels of TNF-α by activating inflammatory signaling pathways, leading to chronic liver disease (32, 33). In contrast, SCFA- and butyrate-producing bacteria, such as *Lactobacillus* and *Flavonifractor*, inhibit harmful bacteria and promote balance in the gut microbiota (33, 34). We found that, regardless of whether mice were suffering from acute liver injury or chronic liver disease, the abundance of some probiotic genera decreased while other symbiotic bacteria increased. In this study, we found that intake of goats’ milk could prevent imbalance in the gut microbiota by promoting the probiotics and reducing harmful bacteria in the gut microbiota.

We also found that there were links amongst goats’ milk consumption, metabolism of the gut microbiota, and biochemical indices. Abundance of the drug metabolism-cytochrome P450 was lower in the gut microbiota of the goats’ milk group of mice than in the model group. This is consistent with down-regulation of the mRNA expression of *CYP2E1* (CYP2E1 is a subtype of CYP450), which we observed in the liver tissue of mice in the goats’ milk group. We also demonstrated that the gut microbiota in the goats’ milk group of mice was involved in amino acid metabolism and energy metabolism in the KEGG pathways, indicating that goats’ milk may provide the energy source required for regulating and improving immunity. This is consistent with our observations on the indicators of liver tissue damage in the goats’ milk group of mice. The interesting point is that the gut microbiota of the goats’ milk group of mice was involved in bile secretion through the KEGG pathways and may thus protect the liver by regulation of bile secretion.

Goats’ milk is an important nutrient source, but the beneficial effects of goats’ milk on organisms with acute hepatic injury induced by CCl_4_ were previously unknown. In our research, we found that consumption of goats’ milk could protect mice from CCl_4_-induced acute hepatic injury and also improved the gut microbiota imbalance caused by CCl_4_. To the best of our knowledge, this is the first time that the hepatoprotective effects of goats’ milk have been demonstrated; goats’ milk has the potential to be developed as a new prospective functional food for alleviation of liver injury.

## Ethics Statement

Animal experiments were performed in accordance with the guidelines of Shaanxi Normal University Scientific Ethics Committee.

## Author Contributions

JZ and YS contributed to the experimental design and data analysis. ZW and DH performed the experiments. YS and ZW wrote the article.

## Conflict of Interest Statement

The authors declare that the research was conducted in the absence of any commercial or financial relationships that could be construed as a potential conflict of interest.

## Supplementary Material

Table S1 The abundances of the metabolic pathways (Level 3) in gut microbiota of the four groups of mice (UPrev, MPrev, GPrev and CPrev).
